# Supplementary material for: Diet Diversity of the Fluviatile Masu Salmon, Oncorhynchus masou (Brevoort 1856) Revealed via Gastrointestinal Environmental DNA Metabarcoding and Morphological Identification of Contents
Source: Biology (Basel). 2024 Feb 18;13(2):129. doi: 10.3390/biology13020129 (PMC10887057; doi:10.3390/biology13020129)
Supplement: Supplementary file 1 [file biology-13-00129-s001.zip › biology-2824926-supplementary.pdf]

Supplemental Information for:

Diet diversity of the fluviatile masu salmon revealed by gastrointestinal tract environmental DNA metabarcoding and contents morphological identification

Lijuan Li [1], Xuwang Yin [2], Qianruo Wan [1], Dilina Rusitanmu [1] and Jie Han [1,*]

1 Ministry of Education Key Laboratory for Biodiversity Science and Ecological Engineering, College of Life Sciences, Beijing Normal University, No. 19 XinJieKouWai Avenue, Beijing 100875, P.R. China
2 Liaoning Provincial Key Laboratory for Hydrobiology, College of Fisheries and Life Science, Dalian Ocean University, NO. 52 HeiShiJiao Avenue, Dalian 116000, P.R. China
* Correspondence: jiehan@bnu.edu.cn

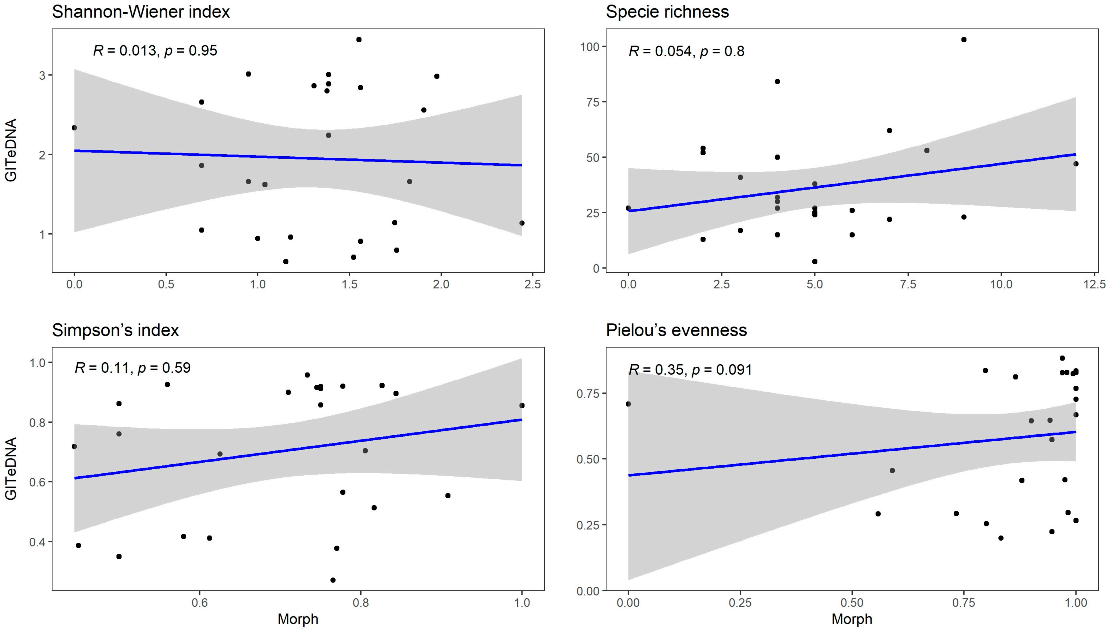

Figure S1 Correlations between diet diversity indices obtained from morphological observation and GITeDNA metabarcoding (*n*=24).

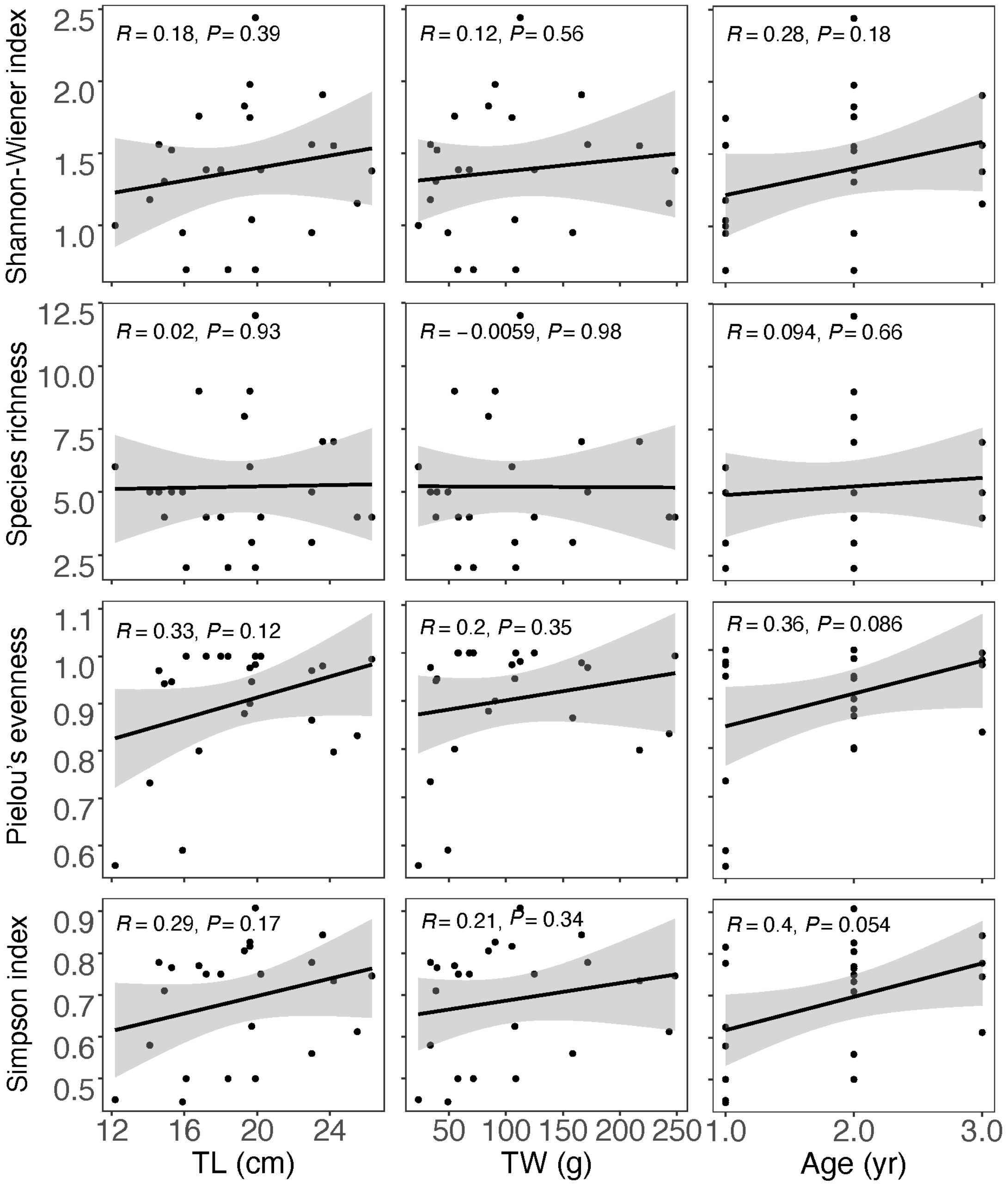

Figure S2 Correlations between body size, age and diet diversity indices of Masu salmon calculated using morphological observation data (TL, total length; TW, total weight; n=24).

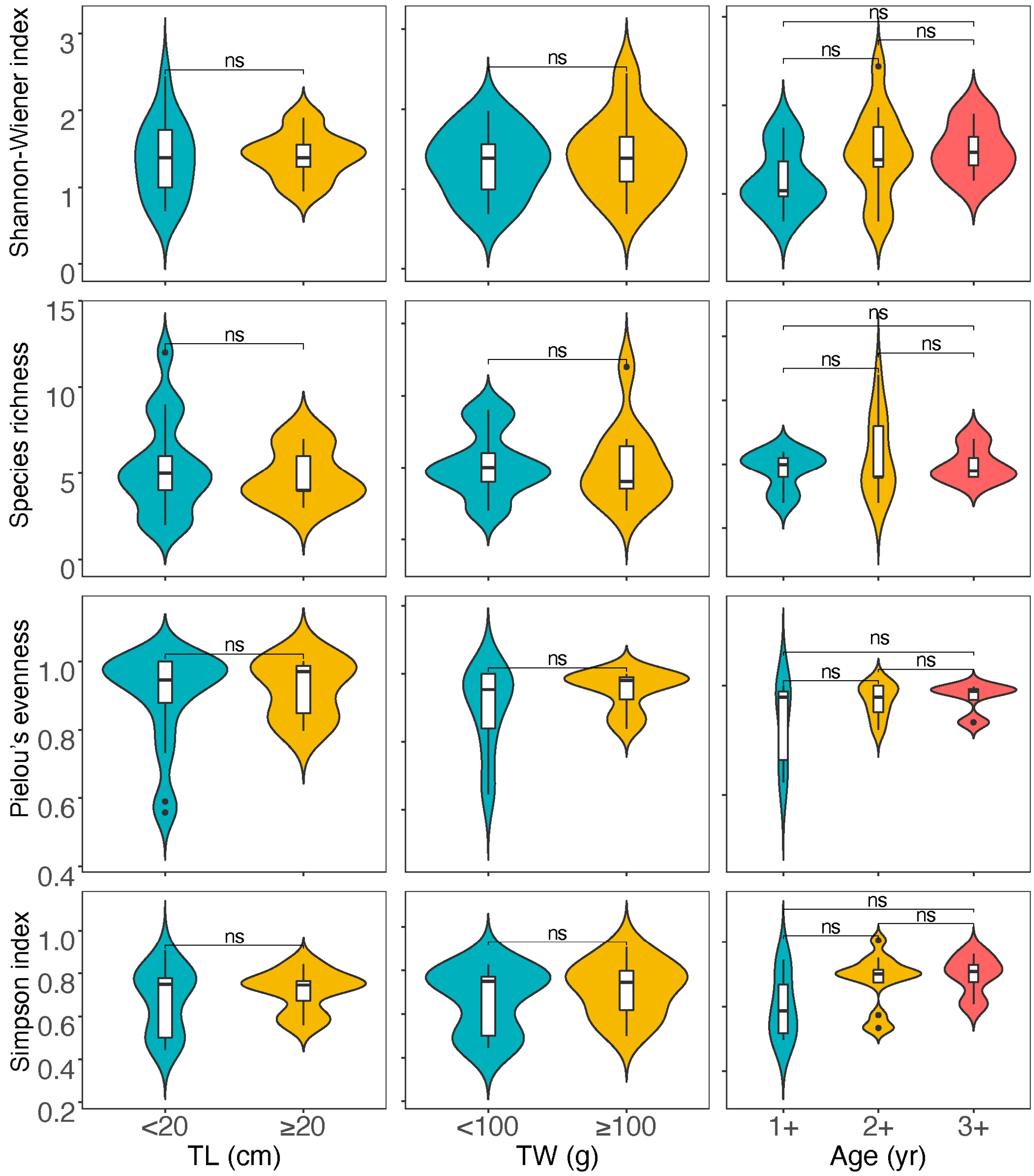

Figure S3 Comparison of diet diversity indices of different size and age groups of Masu salmon (TL, total length; TW, total weight; ns $P \geqslant 0.05$; n=24).

Supplementary Tables

Table S1 Oligo DNA tags attached to the PCR primers which amplifying metabarcode of Masu salmon gastrointestinal tract environmental DNA (GITeDNA).

ccaatgaa	cctgacca	ccatgtca
cccgaatc	ccacagtg	ccgtagag
ccagagac	ccgccaat	ccgtccgc
ccttcgga	cccagatc	ccgtgaac
cccgacgt	ccacgtga	ccgtttcg
ccctcatg	ccgatcag	cccgtacg
cctgtata	cctagctt	ccgagtgg
ccatcacg	ccctggta	ccggtagc
ccctatgt	ccagtcaa	ccactgat
ccttaggc	ccagttcc	ccatgagc
ccattcct		

Table S2 Types of prey and their frequencies of the fluviatile Masu salmon based on data from morphological observation (Morph) and GITeDNA metabarcoding.

Phylum	Class	Order	Family	Genus	Specie	T/A	Frequency of occurance	
							Morph (%)	GITeDNA (%)
Annelida	Clitellata	Arhynchobdellida	Haemopidae	*Haemopis*	spp.*	A	0.336	8.311
		Haplotaxida	Lumbricidae	*Dendrodrilus*	*Dendrodrilus rubidus*	T	-	0.028
		Rhynchobdellida			sp.	A	-	0.014
Arthropoda	Arachnida	Araneae			spp.	T	0.671	0.016
					sp.	T	-	0.041
			Dictynidae		sp.	T	-	0.005
			Philodromidae	*Philodromus*	*Philodromus cespitum*	T	-	3.316
		Sarcoptiformes	Nanhermanniidae		sp.	T	-	0.010
		Trombidiformes			sp.	T	-	0.110
			Pionidae		sp.	T	-	0.009
	Insecta	Coleoptera			spp.	T	1.007	0.227
			Cerambycidae		spp.	T	1.007	0.069
			Cerambycidae	*Leptura*	sp.	T	-	0.016
			Cerambycidae	*Leptura*	*leptura ochraceofasciata*	T	0.671	-
			Chrysomelidae		spp.	T	1.007	0.473
			Chrysomelidae	*Chrysomela*	sp.	T	-	0.034
			Chrysomelidae	*Chrysomela*	*Chrysomela vigintipunctata*	T	0.336	0.043
			Chrysomelidae	*Galerucella*	sp.	T	-	0.118
			Chrysomelidae	*Phratora*	*Phratora laticollis*	T	-	0.006
			Chrysomelidae	*Plagiosterna*	*Plagiosterna aenea*	T	1.678	0.021
			Coccinellidae	*Chilocorus*	sp.	T	-	0.114
			Elateridae	*Limoniscus*	*Limoniscus violaceus*	T	-	0.007
			Scarabaeidae		spp.	T	0.336	-
			Scarabaeidae	*Anomala*	*Anomala dubia*	T	-	0.004
			Spercheidae	*Spercheus*	sp.	A	-	0.406
		Diptera			sp.	-	-	4.209
			Calliphoridae	*Lucilia*	*Lucilia sericata*	T	-	0.005
			Calliphoridae	*Protophormia*	*Protophormia terraenovae*	T	-	0.015
			Cecidomyiidae		sp.	T	-	0.021
			Chironomidae		spp.	A	8.054	0.664
			Chironomidae		sp.	A	-	0.307
			Chironomidae	*Belgica*	*Belgica antarctica*	A	-	0.016

Chironomidae	*Cladotanytarsus*	sp.	A	-	0.016
Chironomidae	*Corynoneura*	sp. 1	A	-	0.009
Chironomidae	*Corynoneura*	sp. 2	A	-	0.018
Chironomidae	*Corynoneura*	sp. 3	A	-	0.810
Chironomidae	*Cricotopus*	sp. 1	A	-	0.659
Chironomidae	*Cricotopus*	sp. 2	A	-	5.732
Chironomidae	*Cricotopus*	*Cricotopus annulator*	A	-	0.009
Chironomidae	*Cricotopus*	*Cricotopus bicinctus*	A	-	0.006
Chironomidae	*Cricotopus*	*Cricotopus perniger*	A	-	0.373
Chironomidae	*Cricotopus*	*Cricotopus rufiventris*	A	-	0.003
Chironomidae	*Cricotopus*	*Cricotopus sylvestris*	A	-	0.007
Chironomidae	*Cricotopus*	*Cricotopus tremulus*	A	-	0.083
Chironomidae	*Cricotopus*	*Cricotopus triannulatus*	A	-	0.079
Chironomidae	*Diamesa*	sp. 1	A	-	0.130
Chironomidae	*Diamesa*	sp. 2	A	-	0.003
Chironomidae	*Gymnometriocnemus*	*Gymnometriocnemus brumalis*	A	-	0.027
Chironomidae	*Halocladius*	*Halocladius variabilis*	A	-	0.002
Chironomidae	*Micropsectra*	*Micropsectra lacustris*	A	-	0.010
Chironomidae	*Micropsectra*	*Micropsectra logani*	A	-	0.077
Chironomidae	*Nanocladius*	sp.	A	-	1.064
Chironomidae	*Orthocladius*	spp.*	A	9.396	0.012
Chironomidae	*Orthocladius*	*Orthocladius oliveri*	A	-	0.109
Chironomidae	*Orthocladius*	sp.	A	-	0.383
Chironomidae	*Pagastia*	*Pagastia orthogonia*	A	-	0.006
Chironomidae	*Phaenopsectra*	sp.	A	-	0.021
Chironomidae	*Polypedilum*	sp. 1	A	-	0.009
Chironomidae	*Polypedilum*	sp. 2	A	-	2.594
Chironomidae	*Polypedilum*	*Polypedilum albicorne*	A	-	0.187
Chironomidae	*Rheocricotopus*	*Rheocricotopus effusus*	A	-	0.037
Chironomidae	*Saetheria*	sp.	A	-	0.098
Chironomidae	*Tanytarsus*	sp. 1	A	-	0.139
Chironomidae	*Tanytarsus*	sp. 2	A	-	0.014
Chloropidae	*Elachiptera*	sp.	T	-	0.421
Culicidae	*Culex*	spp.	A	6.711	-

	Culicidae	*Culex*	*Culex pipiens*	A	-	0.056
	Dolichopodidae	*Dolichopus*	sp.	T	-	0.093
	Drosophilidae		sp.	T	-	0.038
	Drosophilidae	*Drosophila*	sp.	T	-	0.100
	Drosophilidae	*Scaptomyza*	*Scaptomyza pallida*	T	-	0.007
	Empididae		sp. 1	A	-	0.080
	Empididae		sp. 2	A	-	0.058
	Empididae	*Rhamphomyia*	sp.	A	-	0.022
	Muscidae		sp.	T	-	0.002
	Muscidae	*Helina*	sp.	T	-	0.373
	Muscidae	*Limnophora*	sp.	T	-	0.019
	Mycetophilidae	*Mycetophila*	*Mycetophila strigatoides*	T	-	0.014
	Phoridae		sp.	T	-	0.002
	Psychodidae	*Lutzomyia*	*Lutzomyia anduzei*	T	-	9.098
	Sciaridae		sp.	T	-	0.016
	Simuliidae	*Simulium*	spp.*	A	6.376	4.304
	Simuliidae	*Simulium*	sp.	A	-	0.362
	Stratiomyidae		spp.	A	1.342	-
	Tabanidae		sp.	A	-	0.004
	Tabanidae	*Chrysops*	sp.	A	-	0.004
	Tabanidae	*Hybomitra*	*Hybomitra lasiophthalma*	A	-	0.021
	Tachinidae		sp.	T	-	0.004
	Tachinidae	*Medina*	sp.	T	-	0.012
	Tachinidae	*Trichopoda*	sp.	T	-	0.003
	Tephritidae	*Anastrepha*	sp.	T	-	0.007
	Tephritidae	*Anastrepha*	*Anastrepha obliqua*	T	-	0.130
	Tephritidae	*Rhagoletis*	sp.	T	-	0.009
	Tipulidae	*Tipula*	sp.	A	-	0.008
Ephemeroptera			spp.	A	5.034	0.187
	Baetidae		sp.	A	-	0.002
	Baetidae	*Baetis*	*Baetis phoebus*	A	-	0.022
	Ephemerellidae		spp.	A	2.013	-
	Ephemerellidae	*Ephemerella*	*Ephemerella dorothea*	A	-	0.002
	Ephemerellidae	*Drunella*	*drunella trispina*	A	0.336	-
	Ephemerellidae	*Serratella*	spp.	A	1.007	-
	Ephemerellidae	*Serratella*	*Serratella ignita*	A	-	0.015
	Heptageniidae	*Epeorus*	sp.	A	-	0.010
	Heptageniidae	*Rhithrogena*	sp.	A	0.336	0.014
	Leptophlebiidae	*Austrophlebioides*	sp.	A	-	0.044
	Potamanthidae	*Potamanthus*	*Potamanthus tongitibius*	A	5.369	15.759
Hemipter			spp.	T	0.671	-

a						
	Aphrophoridae	*Aphrophora*	spp.*	T	1.678	1.018
	Aphrophoridae	*Aphrophora*	sp.	T	-	0.357
	Miridae		spp.	T	0.336	-
	Miridae	*Poecilocapsus*	*Poecilocapsus lineatus*	T	-	0.077
	Pentatomidae		spp.	T	1.007	0.037
Hymenoptera			sp.	T	-	0.006
	Braconidae	*Lytopylus*	sp.	T	-	0.004
	Cimbicidae		sp.	T	-	0.004
	Formicidae	*Lasius*	sp.	T	-	0.183
	Formicidae	*Lasius*	*Lasius niger*	T	16.443	0.982
	Ichneumonidae		spp.	T	3.691	0.006
Lepidoptera			sp.	T	-	10.241
	Depressariidae	*Agonopterix*	sp.	T	-	0.281
	Erebidae		sp. 1	T	-	0.207
	Erebidae		sp. 2	T	-	0.009
	Erebidae	*Catocala*	sp.	T	-	0.004
	Gelechiidae	*Ardozyga*	sp.	T	-	0.022
	Geometridae		sp.	T	-	2.861
	Nymphulinae		spp.	A	0.336	-
	Oecophoridae		sp.	T	-	0.034
	Sphingidae		spp.	T	0.336	11.006
	Sphingidae	*Amphion*	*Amphion floridensis*	T	-	0.033
	Sphingidae	*Hemaris*	*Hemaris fuciformis*	T	-	0.017
	Sphingidae	*Smerinthus*	sp.	T	-	0.004
	Sphingidae	*Smerinthus*	*Smerinthus cerisyi*	T	-	0.045
Mecoptera			sp.	T	-	0.007
Odonata			spp.	A	0.671	2.244
	Gomphidae	*Gomphidae*	*nihonogomphus ruptus*	A	0.336	-
	Coenagrionidae		sp.	A	-	0.237
Orthoptera	Acrididae		spp.	T	0.671	0.009
Plecoptera			spp.	A	1.007	0.005
Plecoptera			sp.	A	-	0.135
	Capniidae		sp.	A	-	0.141
	Perlodidae		spp.	A	0.671	-
	Perlodidae	*Arcynopteryx*	*Arcynopteryx compacta*	A	-	0.005
	Perlodidae	*Isoperla*	spp.*	A	2.013	0.013
	Perlodidae	*Megarcys*	*Megarcys watertoni*	A	-	0.228
	Perlodidae	*Skwala*	sp.	A	-	0.178
	Pteronarcyidae	*Pteronarcys*	spp.*	A	5.369	0.007
	Taenioptery	*Taenionem*	spp.*	A	0.336	0.059

			gidae	*a*				
		Trichoptera			spp.	A	2.349	0.039
			Brachycentridae	*Brachycentrus*	sp.	A	-	0.004
			Glossosomatidae	*Glossosoma*	sp.	A	-	0.261
			Goeridae	*Goera*	sp.	A	-	0.147
			Hydropsychidae	*Hydropsyche*	spp.*	A	0.336	0.150
			Limnephilidae		sp.	A	-	0.027
			Phryganeidae	*Agrypnia*	*Agrypnia colorata*	A	-	5.450
			Stenopsychidae	*Stenopsyche*	spp.*	A	0.336	0.184
Chordata	Actinopterygii	Cypriniformes			spp.	A	6.711	-
			Cyprinidae	*Phoxinus*	*Phoxinus phoxinus*	A	0.671	0.026
			Cyprinidae	*Rhynchocypris*	sp.	A	-	0.019
			Nemacheilidae	*Barbatula*	spp.*	A	1.007	0.002
		Salmoniformes	Salmonidae	*Brachymystax*	sp.	A	-	0.304
			Salmonidae	*Brachymystax*	*Brachymystax lenok*	A	-	0.002

"sp." and "spp." stand for single and several unknown or not specified species, respectively.

"spp.*" indicates that only one species identified by morphological observation which may be different from that identified by GITeDNA barcoding.

A, aquatic prey; T, terrestrial prey.

Table S3 Diet diversity of the fluviatile Masu salmons.

Fish code	TW	TL	Age	Debris	Morphological observation				GITeDNA metabarcoding			
	(g)	(cm)	(year+)	RV (%)	Shannon	Richness	Simpson	Evenness	Shannon	Richness	Simpson	Evenness
M2	171.6	23.0	3	3	1.561	5	0.778	0.970	2.841	25	0.921	0.883
M3	178.3	22.9	3	10	0.000	1	0.000	NA	2.197	28	0.830	0.659
M4	108.6	19.9	2	5	0.693	2	0.500	1.000	2.662	54	0.862	0.667
M6	84.7	19.3	2	40	1.828	8	0.806	0.879	1.660	53	0.704	0.418
M7	166.3	23.6	3	25	1.906	7	0.844	0.980	2.560	22	0.896	0.828
M8	243.1	25.5	3	13	1.154	4	0.612	0.832	0.655	27	0.412	0.199
M9	124.9	20.2	2	15	1.386	4	0.750	1.000	2.244	15	0.858	0.829
M10	107.8	19.7	1	60	1.040	3	0.625	0.946	1.623	17	0.693	0.573
M11	214.9	23.3	2	empty	NA	NA	NA	NA	2.336	27	0.855	0.709
M13	90.5	19.6	2	30	1.977	9	0.826	0.900	0.908	3	0.565	0.826
M14	33.9	14.6	1	20	1.561	5	0.778	0.970	2.984	103	0.923	0.644
M15	158.5	23.0	2	10	0.950	3	0.560	0.865	3.012	41	0.925	0.811

M16	217.1	24.2	2	40	1.553	7	0.734	0.798	3.446	62	0.958	0.835
M17	248.2	26.3	3	0	1.378	4	0.746	0.994	2.800	30	0.917	0.823
M18	140.1	22.0	2	6	0.000	1	0.000	NA	3.116	38	0.945	0.857
M20	190.6	24.3	2	20	0.000	1	0.000	NA	3.236	62	0.937	0.784
M21	107.1	20.1	3	5	0.000	1	0.000	NA	2.003	14	0.819	0.759
M22	67.9	18.0	2	10	1.386	4	0.750	1.000	2.890	32	0.919	0.834
M23	57.8	16.1	2	5	0.693	2	0.500	1.000	1.048	52	0.350	0.265
M24	49.1	15.9	1	15	0.950	5	0.444	0.590	1.657	38	0.719	0.456
M25	71.4	18.4	1	5	0.693	2	0.500	1.000	1.862	13	0.760	0.726
M26	112.5	19.9	2	30	2.441	12	0.908	0.982	1.137	47	0.553	0.295
M27	124.6	20.2	1	40	0.000	1	0.000	NA	0.004	5	0.001	0.002
M28	58.3	17.2	2	20	1.386	4	0.750	1.000	3.003	50	0.912	0.768
M30	99.1	19.3	2	50	0.000	1	0.000	NA	1.549	29	0.642	0.460
M31	105.3	19.6	1	5	1.748	6	0.816	0.976	1.140	15	0.513	0.421
M32	33.7	14.1	1	20	1.179	5	0.580	0.732	0.962	27	0.417	0.292
M33	23.2	12.2	1	50	1.000	6	0.450	0.558	0.947	26	0.387	0.291
M34	39.6	15.3	2	30	1.523	5	0.765	0.946	0.709	24	0.271	0.223
M36	54.9	16.8	2	40	1.758	9	0.770	0.800	0.797	23	0.378	0.254
M37	38.6	14.9	2	10	1.306	4	0.710	0.942	2.866	84	0.900	0.647
Mean ±sd	113.61 ±64.93	19.66 ±3.6	1.94 ±0.68	21.07 ±16.40	1.10 ±0.68	4.37 ±2.79	0.55 ±0.31	0.90 ±0.13	1.96± 0.95	35.03±22.20	0.70± 0.25	0.58± 0.25

TW, total weight; TL, total length; RV, the relative volume of the debris to that of gastrointestinal tract.

Table S4. Diet diversity in the divided fluviatile Masu salmon groups.

Data	Group	*n*	Shannon-Wiener index	Species richness	Pielou's evenness	Simpson index
GITeDNA (Mean±sd)	<20 cm	18	1.69±0.83	38.33±25.28	0.50±0.21	0.64±0.22
	≥20 cm	13	2.34±1.00	30.46±16.95	0.69±0.27	0.79±0.28
	<100 g	14	1.70±0.88	39.79±27.05	0.51±0.23	0.63±0.24
	≥100 g	17	2.18±0.97	31.12±17.12	0.64±0.26	0.76±0.26
	age 1+	8	1.14±0.59	0.45±0.26	18.00±11.80	0.51±0.25
	age 2+	17	2.28±0.93	0.61±0.23	46.82±22.73	0.76±0.23
	ag e 3+	6	2.18±0.82	0.69±0.25	24.33±5.75	0.80±0.19
Morph (Mean±sd)	<20cm	17	1.36±0.49	5.35±2.78	0.90±0.14	0.68±0.15
	≥20cm	7	1.41±0.31	4.86±1.57	0.92±0.09	0.72±0.10
	<100g	13	1.33±0.41	5.23±2.28	0.87±0.16	0.66±0.15
	≥100g	11	1.44±0.49	5.18±2.79	0.94±0.07	0.72±0.13

age 1+	7	1.17±0.37	4.57±1.51	0.82±0.19	0.60±0.15
age 2+	13	1.45±0.50	5.62±3.10	0.93±0.08	0.72±0.12
ag e 3+	4	1.50±0.32	5.00±1.41	0.94±0.08	0.74±0.10
